# An Evaluation of Hepatitis C Screening in Infants and Children Born to Seropositive Mothers in Saint John, New Brunswick

**DOI:** 10.7759/cureus.17377

**Published:** 2021-08-23

**Authors:** Sarah Gander, Angela Morris, Stefanie Materniak

**Affiliations:** 1 Pediatrics, Saint John Regional Hospital, Saint John, CAN; 2 Pediatrics, Dalhousie Medicine New Brunswick, Saint John, CAN; 3 Infectious Disease, Horizon Health, Saint John, CAN

**Keywords:** hepatitis c virus, hcv, vertical transmission, screening, risk factors

## Abstract

Background: The primary route of hepatitis C virus (HCV) infection in children is vertical transmission, from mother to fetus in utero. There is a lack of data on the prevalence of pediatric HCV acquired through vertical transmission in Saint John, New Brunswick. Furthermore, what risk factors may be associated with an increased likelihood for a child born to an HCV-seropositive mother should be known to direct screening practices.

Methods: A retrospective chart review of the active charts from the local HCV clinic, the Centre for Research, Education & Clinical Care of At-Risk Populations (RECAP), identified HCV-seropositive women who had children at-risk of HCV through vertical transmission. Sociodemographic information and various risk factors were collected, including maternal HCV genotype, non-prescription drug use subcategorized into intravenous drug use and snorting, transfusion history, involvement in opiate substitution therapy, postal code as a proxy for socioeconomic status, and issues of custodianship within the family. A 2 x 2 chi-square analysis was conducted to assess the frequency of HCV screening for children by the presence or absence of familial custodianship issues.

Results: In total, data from 62 HCV-seropositive women and 123 infants and children at-risk for HCV were included in this study. HCV status at the time of pregnancy revealed 18 (14.6%) with a positive HCV screen, 14 (11.4%) with a positive viral load, and 91 (74.0%) with unknown status. A total of 30 children (24.4%) had HCV screening performed, of which three (10.0%) were HCV-antibody positive and had a detectable viral load. Results of the chi-square analysis indicated that issues of custodianship had no significant influence on child screening rates.

Conclusion: Overall, this study highlighted the inconsistent screening practices of children at-risk for HCV through vertical transmission, as well as the need for improvement in chart documentation and follow-up. Clinicians and researchers should focus their efforts toward proactively identifying children at-risk for HCV through vertical transmission. This could involve screening during pregnancy and subsequent follow-up, or at other points of contact with the healthcare system, such as parental involvement with opioid substitution therapy or well-child visits. Implementation of a targeted screening program could be considered in urban centers similar to the one in this study to connect at-risk populations with essential medical and community services.

## Introduction

Hepatitis C Virus (HCV) is a global health concern that has significant health consequences, including chronic liver disease, cirrhosis, hepatocellular carcinoma, and liver transplantation [1]. Approximately 2.5% of the world's population is infected with HCV, with rates between 1% and 8% in pregnant women and between 0.05% and 5% in children [2]. The World Health Organization (WHO) aims to eliminate HCV infection as a public health threat by 2030 [3,4].

Vertical transmission of hepatitis C

HCV is highly transmissible and is spread through contact with infected blood. Mother-to-child transmission in utero, or vertical transmission, is the primary route of HCV infection in children. A recent meta-analysis estimated that the vertical transmission rate of mother-to-child is 5.8%, indicating greater than one in every 20 children delivered by HCV-seropositive women could be infected [5]. Due to the increasing rates of new HCV infection, secondary to intravenous drug use and the opioid epidemic, more women of childbearing age could be transmitting HCV to their children [6]. A population-based retrospective study found that the prevalence of HCV-infected pregnant women increased by 60% between 2006 and 2014. Another study found a doubling effect with HCV rates between 2009 and 2014 in pregnant women reaching 3.4 per 1000 live births nationwide in the United States [7-8]. Notably, this number could be underreported due to the asymptomatic presentation of HCV, which also presents a concern as many women may be unaware of their HCV status, the risk of transmission, or further testing recommendations for their children.

Screening of hepatitis C

HCV infections can be asymptomatic for years and often remain undiagnosed; thus, infected individuals can act as reservoirs for further infection. Current guidelines from the Canadian Association for the Study of the Liver recommend risk factor-based screening for children born to HCV-seropositive mothers [9]. However, such screening is not always done. A retrospective cohort study of infants exposed to HCV in the United States found only 23% of 4072 infants were screened [10]. Another study on HCV screening rates at a methadone program in Australia found that while 70% of pregnant women were seropositive for HCV, less than 20% of their offspring were examined [11]. This is concerning as studies have shown that approximately 80% of perinatally-acquired HCV develops into a chronic infection, which has the potential to lead to various health concerns, including chronic liver disease, cirrhosis, hepatocellular carcinoma, and liver transplantation [1,12].

The vertical transmission rate of mother-to-child is approximately 5.8%, and a higher maternal viral load has been proven to increase the risk of infection [5,13]. Screening and diagnosis of HCV infection in infants and children cannot be completed at birth as maternal HCV immunoglobulin G antibodies are present in infants until approximately 18 months of life [14]. Furthermore, children have high rates of spontaneous clearance, with studies showing a spontaneous clearance rate ranging from 21% to 25% [12,13]. Any screening tests completed before maternal antibodies clear or before spontaneous clearance could lead to false-positive results. Current standards for screening test for serum anti-HCV antibodies and alanine aminotransferase (ALT) activity in the blood, then if positive, HCV can be diagnosed via HCV-ribonucleic acid (RNA) detection by polymerase chain reaction (PCR) [15].

Risk factors and screening status

Current guidelines from the Canadian Association for the Study of the Liver recommend risk factor-based screening instead of universal screening for HCV during pregnancy, although it has been found to only identify half of the women infected with HCV [9]. To identify infants at risk of vertical transmission of HCV, the infection must first be identified in the mothers. Various factors may prompt providers to screen mothers and infants for HCV infection. Most notably, a history of intravenous drug use or opioid use disorder has been positively associated with pediatric HCV screening [7,12]. Involvement in HIV or methadone maintenance therapy at chronic care clinics and attendance of well-child visits improved screening of at-risk infants [10, 16]. 

The current study

Previous research supports the role of screening for infants born to at-risk mothers. In Saint John, New Brunswick, and the surrounding area, there is limited data regarding the rate of hepatitis C in the general population. There are several anecdotal and formal observations of neonatal screening opportunities being missed or physicians not aware of when to implement a screening initiative for these at-risk infants. The current study performed a retrospective chart review of all active charts at the Centre for Research, Education & Clinical Care of At-Risk Populations (RECAP) to identify women with HCV seropositivity who have had children who are at-risk for HCV through vertical transmission. RECAP is a not-for-profit organization that delivers care to individuals positive and/or at-risk for HCV. It is the only HCV clinic in Saint John and the surrounding area providing community and healthcare services for HCV-positive individuals.

The Objectives for the Current Study

Evaluate the current rates of screening for at-risk infants and children born to HCV-positive mothers at the Centre for Research, Education & Clinical Care of At-Risk Populations (RECAP).

Understand the background characteristics (e.g. viral load, genotype) and risk factors (e.g. non-prescription drug use excluding cannabis subcategorized into intravenous drug use and snorting, transfusion history, opiate substitution therapy, and income quintile determined by postal code analysis) for HCV-positive women who have babies who are at-risk for HCV through vertical transmission.

Determine if issues of custodianship impact the rate at which at-risk infants and children are screened for HCV.

## Materials and methods

Participants

Infants and children born to HCV-positive mothers were identified through retrospective chart review from active charts of seropositive women at RECAP. Data were collected from the charts of 62 mothers for a total of 123 children. Notably, any infants under 18 months would not yet be eligible for screening and were therefore excluded. However, if the child’s birth date was not indicated, the mother’s HCV status at pregnancy was recorded as "unknown".

Materials

Data for the current study was extracted from active charts at RECAP. The data collected included the mother’s date of birth (month/year), HCV testing date(s) and viral load(s) as measured by serum RNA level, genotype (1a/1b/2/3/4/5/6), and risk factors, including non-prescription drug use (excluding cannabis) subcategorized into intravenous drug use and snorting, transfusion history, opiate substitution therapy, and postal code data which was used as a proxy for socioeconomic status. Finally, custodianship issues were recorded based on data provided in the mother’s chart; this could involve mention of custodianship cases, children living with other family members, foster care or adoption, and jail time on the part of the mother. The initial intention was to record on a per-child basis; however, due to lack of information on custodianship issues, it was recorded based on maternity. Variables specific to the children included the number of children born to each mother, date of birth when available (month/year), screening status (yes/no) and the date of screening, the screening results, and the testing rationale (i.e. screening/clinically indicated). For this study's purpose, if the child’s screening status was not indicated in the RECAP charts, it was assumed that screening had not been done. As this research was not originally intended, ethical approval for secondary use of data was granted from the Horizon Health Network’s research ethics board (IRB approval number: 100234). Finally, statistical analysis was conducted using SPSS (IBM Corp. Released 2015. IBM SPSS Statistics for Windows, Version 23.0. Armonk, NY: IBM Corp) statistical software.

Procedure

Retrospective chart review of all active charts at RECAP identified women that were HCV-seropositive in a timeframe surrounding their pregnancy and pertinent sociodemographic information was collected including the above risk factors and information on infant and children screening status. Information from the chart review was input into an excel database and appropriately coded for analysis. The data were checked for errors and missing variables. Analysis of postal code data, used as a proxy for socioeconomic status, was conducted using SAS version 9.4 (SAS Institute Inc., Cary, NC, USA) and the most recent postal code conversion file (PCCF+ 7C), which links postal codes to corresponding census data from that area. These data were analyzed in SPSS. Further descriptive statistics were obtained for variables of interest and presented as the frequency and percentage. The data file was then separated into parental and child-centric datasheets for further analysis. Custodianship values were filled in to create matching sample sizes (n = 123) and a 2 x 2 chi-square test was performed assessing screening status by custodianship using an alpha level of 0.05.

## Results

Information on 62 HCV-seropositive mothers and 123 children who were at risk for vertical transmission were collected through retrospective charts from the active charts at RECAP. Table 1 provides the percentage distribution of selected characteristics including HCV genotype, non-prescription drug use, intravenous drug use, snorting, transfusion, opiate substitution therapy, and issues of custodianship. Maternal HCV genotypes included 19 (30.6%) in 1a, 11 (17.7%) in 3a, four (6.5%) in 1b, two (3.2%) in 2a/c, one in (1.6%) 2b, and 25 (40.3%) unknown. Non-prescription drug use was present in 57 (91.9%), four (6.5%) did not report using non-prescription drugs, and one (1.6%) was unknown. Intravenous drug use was present in 55 (88.7%), absent in six (9.7%), and one (1.6%) unknown. Snorting was present in 22 (35.5%), absent in 39 (62.9%), and one (1.6%) unknown. Only one (1.6%) chart documented the contraction of HCV via a blood transfusion. A total of 47 (75.8%) mothers were involved in opiate substitution therapy, 13 (21.0%) were not involved, and two (3.2%) were unknown. Finally, 35 (56.5%) mothers had issues of custodianship, 26 (41.9%) did not, and one (1.6%) was unknown. 

**Table 1:** Frequency and percentage distribution of selected characteristics of mothers who have had children at-risk of vertical transmission of HCV

Demographic (n = 62)		Frequency	Percentage (%)
Participant genotype			
	1a	19	30.6
	1b	4	6.5
	2a/c	2	3.2
	3a	11	17.7
	2b	1	1.6
	Unknown	25	40.3
Non-prescription drug use			
	Yes	57	91.9
	No	4	6.5
	Unknown	1	1.6
Intravenous drug use			
	Yes	55	88.7
	No	6	9.7
	Unknown	1	1.6
Snorting			
	Yes	22	35.5
	No	39	62.9
	Unknown	1	1.6
Transfusion			
	Yes	1	1.6
	No	60	96.8
	Unknown	1	1.6
Opiate substitution therapy			
	Yes	47	75.8
	No	13	21.0
	Unknown	2	3.2
Custodianship			
	Yes	35	56.5
	No	26	41.9
	Unknown	1	1.6

Table 2 indicates the area-based neighbourhood income quintile (QABTIPPE) associated with each maternal postal code. Income quintiles are based on the before-tax income per single person equivalent (BTIPPE) in that neighbourhood according to census data. The results illustrate that 64.2% of the participants lived in an area associated with one of the two lower-income quintiles.

**Table 2:** Area-based neighbourhood income quintile (QABTIPPE) associated with maternal postal code

		Frequency	Percent	Valid Percent	Cumulative Percent
Valid	1.00	27	50.9	50.9	50.9
	2.00	7	13.2	13.2	64.2
	3.00	9	17.0	17.0	81.1
	4.00	7	13.2	13.2	94.3
	5.00	3	5.7	5.7	100.0
	Total	53	100.0	100.0	

HCV status at the time of pregnancy (n = 62 mothers, 123 pregnancies) revealed 18 (14.6%) with a positive HCV screen, 14 (11.4%) with a positive viral load, and 91 (74.0%) with unknown status (Table 3). A total of 30 infants had HCV screening performed (n = 123), of which three (10.0%) were HCV-antibody positive and had a detectable viral load.

**Table 3:** Summary of child’s HCV screening status and results, including the maternal HCV status during pregnancy

Demographic		Frequency	Percentage (%)
Mother’s pregnancy HCV status			
	Positive screen	18	14.6
	Positive viral load	14	11.4
	Tests unknown at the time of pregnancy	91	74.0
Child’s screening status			
	Yes	30	24.4
	No	93	75.6
Child’s screening results			
	Positive	3	10.0
	Negative	27	90.0

The presence or absence of custodianship issues was found to be non-significant on chi-square analysis (Table 4).

**Table 4:** Chi-square analysis assessing the frequency of HCV screening for children by the presence or absence of custodianship issues related to their mothers a = Alpha level of 0.05

Demographic		Frequency		p-value^a^
Custodianship		Children screened	Children not screened	0.778
	Issues – not all children in the care of the mother	19	55	
	No issues – all children in the care of the mother	11	36	

## Discussion

Overall, this study highlights the inconsistent screening practices of infants exposed to HCV through vertical transmission as well as the need for improvement in chart documentation and follow-up. The current rate of screening at-risk infants and children born to HCV-positive mothers at RECAP is only a quarter (24.4%) of the children exposed to HCV through vertical transmission. Of those screened, three (10%) were HCV-antibody positive and had a detectable viral load and it is of concern that children not receiving screening may be going undiagnosed and untreated. The lack of screening could be due to a variety of factors encompassing the challenges in screening as well as a lack of data collection.

A recent study on the challenges of HCV in infants, children, and adolescents found that actively identifying mothers at-risk for HCV effectively captured infants at risk of HCV infection through vertical transmission [17]. However, there was a high “no-show” rate associated with difficulty to follow-up as well as multiple social challenges, including a lack of awareness on the importance of follow-up [17]. This could be further impacted by the 18 months wait time required to screen infants to ensure maternal HCV immunoglobulin G antibodies are no longer present [14]. Additional factors impacting screening included a failure to transfer information regarding maternal HCV diagnosis to the pediatric record as well as a failure to disclose HCV status due to stigmatization or unawareness of infection status [7, 18]. Documentation of pediatric data is not a deliberate step at RECAP, and this perhaps overlooks at-risk infants while actively treating their mothers. This study sought to add to the current literature by looking at issues of custodianship as a factor impacting screening. However, the results were found to be non-significant (p = 0.778). Educational awareness on the importance of screening and proper transfer of information, especially in the context of issues of custodianship for foster families and social workers, is key to ensuring that all at-risk infants are screened appropriately.

There is a need for improvement in chart documentation and follow-up as information regarding infants and children was inconsistently reported in maternal charts at RECAP, probably because it isn't a deliberate step in the documentation. The current study noted that when the age or date of birth of the child was not recorded correlating the viral load during pregnancy, it resulted in a vast majority of the data set having unknown HCV status at the time of pregnancy (74%). Issues of custodianship were infrequently documented on a per-child basis, which resulted in the present study changing the initial intention to instead identify issues of custodianship as a maternal factor. In particular, custodianship may be challenging to report due to frequent changes in the status and because this population can be difficult to engage and follow up. This lack of data has important public health implications as without appropriate screening children may experience a delay in diagnosis and subsequent treatment leading to further health concerns. Notably, other studies have found rates of screening in infants to be as low as 23% despite being exposed to HCV at birth and receiving other well-child services [7,10]. Therefore, awareness of HCV infection and systematic identification of at-risk mothers and infants should be developed.

## Conclusions

Limitations

The study has some limitations. The study collected retrospective data from active charts at RECAP, representing a single medical center, and may have missed HCV-infected mothers whose charts were no longer active as well as HCV-positive women who have not had any involvement with RECAP. It is possible that the HCV transmission rate could be higher among women who have never accessed treatment and that they may have higher viral loads. Other limitations of the study are the lack of data on children in their mothers' charts. In particular, many charts did not reference the child’s date of birth which impacted the ability to cross-reference the mother's HCV status and viral load with their pregnancy. Finally, custodianship was rarely reported, thus leading to an alteration in the methods of this study.

Implications and future directions

Further studies could look into the various points of contact with the health care system for proactive screening opportunities since an untreated HCV infection will lead to significant health consequences including chronic liver disease, cirrhosis, hepatocellular carcinoma, and liver transplantation. One argument made for the elimination of vertical transmission involves implementing universal as opposed to targeted screening during pregnancy since only half of the women who are HCV-positive are identified by screening based on risk factors. The perinatal period offers an excellent opportunity for screening and diagnosis of HCV when women may be motivated to consider curative treatment and reduce the risk of vertical transmission in subsequent pregnancies. Other factors that improved screening of infants pointed to women involved in care for HIV or methadone maintenance therapy. Combining infant programs for HCV screening with chronic care clinics for women could maximize infant HCV follow-up while providing services to women. Good primary care and trust built within individual healthcare providers or a clinic could increase success in the identification and follow-up of at-risk infants. Furthermore, there are observed needs in this population that may lend themselves to the provision of support and compliance with known resources in the community during the time of screening such as vaccination updates and developmental screens.
